# EmergInsight: a real-time dashboard for optimizing emergency care through data visualization and analytics

**DOI:** 10.1093/jamiaopen/ooag008

**Published:** 2026-01-20

**Authors:** Francesco Branda, Vincenzo Andretta, Mohamed Mustaf Ahmed, Antonio Rizzelli, Valentina Cerrone, Giovanni Boccia, Giancarlo Ceccarelli, Fabio Scarpa, Massimo Ciccozzi

**Affiliations:** Unit of Medical Statistics and Molecular Epidemiology, Campus Bio-Medico University of Rome, Rome, 00128, Italy; Department of Medicine, Surgery and Dentistry—Salerno Medical School, University of Salerno, Fisciano, 84084, Italy; Faculty of Medicine and Health Sciences, SIMAD University, Mogadishu, 252, Somalia; Research and Development Manager and Qlik Specialist, Ethica System Srl, Capurso, 70010, Italy; University Clinical Oncology Unit, University Hospital ‘San Giovanni Di Dio e Ruggi D’Aragona’, Salerno, 84131, Italy; Department of Medicine, Surgery and Dentistry—Salerno Medical School, University of Salerno, Fisciano, 84084, Italy; Department of Public Health and Infectious Diseases, University of Rome Sapienza, Rome, 00185, Italy; Department of Biomedical Sciences, University of Sassari, Sassari, 07100, Italy; Unit of Medical Statistics and Molecular Epidemiology, Campus Bio-Medico University of Rome, Rome, 00128, Italy

**Keywords:** emergency department, real-time analytics, healthcare dashboard, decision support systems, health informatics

## Abstract

**Objectives:**

The fragmentation of healthcare data in Italy and the increase in demand in emergency departments (EDs) require innovative solutions to improve operational flows, quality of care, and decision-making. This study presents EmergInsight, a prototype interactive real-time dashboard designed to optimize ED operations through the visualization and analysis of clinical and operational data.

**Materials and Methods:**

EmergInsight was tested on data from the “San Giovanni di Dio e Ruggi d‘Aragona” University Hospital (Salerno, Italy) from 2022 to 2023. The dashboard integrates performance indicators, geospatial maps, trend analysis, and predictive tools, structured into four modules focused on operational efficiency, acuity management, resource utilization, and demand forecasting.

**Results:**

EmergInsight revealed seasonal access patterns, high green code prevalence, early dropouts, and frequent Intensive Short Observation (OBI) use in elderly patients. Geographic maps identified high-care demand areas, while the predictive module provided timely and reliable forecasts of patient access.

**Discussion and Conclusion:**

EmergInsight facilitates proactive ED management, improving resource allocation and supporting data-driven decisions.

## Background and significance

Emergency departments (EDs) globally face increasing pressures that compromise their ability to provide timely and effective care. Key challenges include prolonged wait times for patients, caused by a combination of factors such as increasing access volume, staff shortages, and budget constraints. The consequences of these pressures are significant: they can reduce patient satisfaction,^1^ increase the risk of adverse outcomes, and compromise the overall quality of care. In parallel, the health care sector is experiencing unprecedented growth in data production. From clinical records to diagnostic images, from vital parameters to treatment results, the enormous amount of information available represents a valuable but difficult resource to manage with traditional methods. In this context, data visualization and real-time analytics are emerging as key tools for transforming this complex data into useful, operational knowledge. These approaches enable healthcare professionals to extract relevant insights, improve decision making, and optimize resource and care management in emergency settings. For example, the implementation of interactive dashboards has been shown to effectively support real-time decision-making, improving patient flow and resource management in the ED.^2^

Public health informatics, a discipline that integrates healthcare, data science, informatics, and communication, offers an effective theoretical and practical framework for addressing these challenges. Its main goal is to improve the efficiency, accuracy and delivery of health services, especially in surveillance and emergency response activities. The widespread adoption of electronic health records and other digital data sources now makes it possible to apply health informatics principles in practical ways to improve public health outcomes. One significant example is the use of digital dashboards for visualizing public health data, which has been shown to improve risk communication and situational awareness during the COVID-19 pandemic.^3^ In particular, data visualization plays a key role, allowing complex datasets to be visually represented and facilitating the identification of patterns, trends, and correlations. This process makes data more accessible and interpretable, facilitating more informed clinical decisions, more efficient resource management, and better patient outcomes. Interactive visualization techniques, in particular, allow multiple dimensions of health data to be explored simultaneously, generating insights useful for operational activities in emergency rooms. For example, the use of real-time visualization systems to transfer patient data from the ambulance to the ED can improve clinical preparedness and intervention, optimizing workflow and quality of care.^4^ The urgency to develop such systems is also reinforced by real data from the Italian context. A recent study conducted at Salerno Hospital analyzed the causes of ER overcrowding, highlighting a high incidence of inappropriate access and poor system coordination as major contributors to ER congestion.^5^ These findings further support the need for data-driven tools that can anticipate access trends, improve triage flows, and optimize resource allocation. Main contribution of this paper are: (1) Development of an integrated dashboard that combines real-time analysis and interactive data visualization for monitoring the performance of an emergency room. (2) Implementation of geospatial analysis tools to map emergency room users, improving situational awareness and supporting resource allocation. (3) Demonstration of modular data architecture designed for interoperability and scalability, enabling potential replication across multiple healthcare facilities despite fragmentation in existing electronic health record (EHR) systems.

## Related work

The use of dashboards in EDs has become increasingly important in recent years, as healthcare facilities strive to equip themselves with decision-making tools that support real-time monitoring, patient flow visualization, and resource allocation. Dashboards translate complex sets of clinical and operational data into immediately usable visual representations, facilitating both tactical decisions (eg, bed assignment, waiting times, triage) and strategic decisions (eg, planning, demand forecasting). Various lines of research on ED dashboards have emerged in the literature: (1) real-time visualization and monitoring; (2) prediction and advanced analysis; (3) development of performance indicators and contextualization; (4) fragmented contexts and interoperability.

Some work has focused on how to make key indicators immediately visible within the ED. Yoo et al^6^ have presented the development of a real-time, autonomous dashboard for a Korean ED, designed according to three core principles—“anytime, anywhere, at a glance”; “minimal interruption to workflow”; and “protection of patient privacy.” The system integrates three main functional components: a geographical layout for spatial awareness, a patient-level alert system to highlight critical cases, and real-time summary data for rapid situational assessment. Evaluation results demonstrated good usability, with an average System Usability Scale (SUS) score of 67.6. Participants reported positive feedback regarding enhanced concentration, effective visualization of variability, and the high quality of information provided. Nonetheless, some limitations were noted, particularly concerning divided attention when simultaneously managing multiple information streams. Hamouda et al^7^ have introduced and implemented a comprehensive dashboard solution within a Tunisian university hospital, aiming to support both clinical care and medical training activities. Their approach involved the identification and formal definition of key performance indicators (KPIs) relevant to emergency department operations and academic objectives. Based on these indicators, the authors developed a summary management dashboard intended to assist department leaders in monitoring performance, optimizing resource allocation, and improving decision-making processes. A second line of research concerns the integration of predictive models, advanced analyses, and visualization tools on a regional or supra-hospital scale, with the aim of supporting system decisions and anticipating changes in the demand for care. For example, Hanf et al^8^ have illustrated the development of an interactive online dashboard capable of processing daily data from emergency rooms and emergency medical services across the entire Île-de-France region. The system provides summary statistics and historical trends, seven-day forecasts based on machine learning models, comparisons with previous years, and analyses stratified by age group, thus offering a dynamic and comparative picture of the trend in healthcare demand. This approach allows health authorities to anticipate peaks in demand and optimize resource planning on a territorial scale. Tsai et al^9^ have described the integrated adoption of IoT technologies, artificial intelligence, and clinical dashboards for real-time prediction of unfavorable prognosis in emergency room patients suffering from eight different critical conditions. The proposed system was based on a four-level architecture that combined automated data collection, predictive processing using machine learning models, interactive visualization of results, and clinical decision support. The results showed that the integration of AI and visual analytics tools improved healthcare professionals’ ability to identify risk situations early and intervene proactively.

A systematic review^10^ have analyzed literature published between 2000 and May 2020, identifying how the indicators used in emergency department dashboards were generally grouped into five main categories: quality of care, patient flow, timeliness, costs, and resources. The most common functionalities were reporting, interface customization, alert systems, resource management, and real-time data visualization. The main critical issues were associated with the quality and origin of data sources, integration with other information systems, adaptability to end-user needs, and the choice of the most appropriate indicators to represent ED performance. Another empirical application conducted in Argentina^11^ have illustrated the development and implementation of a quality dashboard for the emergency department, based on indicators defined by an interdisciplinary team. The project involved the creation of a user-centered interface and the use of web-based business intelligence (BI) tools for data management and visualization. The results showed that the system helped identify operational criticalities, including cases of undertriage, thus supporting the continuous improvement of care processes.

A less explored area concerned contexts characterized by fragmented data infrastructures, complex regional systems, and critical phenomena such as emergency room crowding. For example, Martin^12^ have illustrated the development of a dashboard for emergency room crowding in Belgium (Flanders), following a design science approach. Through a Delphi study, the authors identified specific indicators of crowding, which were subsequently implemented using ShinyDashboard in R for real-time visualization and monitoring. More recently, Baan-Kooman et al^13^ have analyzed the adoption of an online dashboard for ambulance diversion in the Netherlands. Although the impact on crowding was limited, the results showed widespread use of the tool in many emergency departments, with a response rate to interviews exceeding 90%. The study also highlighted that crowding is a multifactorial phenomenon, influenced by multiple operational, organizational, and structural determinants.

Overall, the literature highlights that ED dashboards offer established benefits, including greater operational visibility, flow optimization, real-time decision support, targeted indicator selection, and user-centered design. Despite these advantages, significant limitations and gaps remain: many studies are conducted in contexts with mature and well-integrated digital infrastructures, with little attention to interoperability in fragmented systems; evidence on the extension of dashboards to strategic and regional dimensions remains limited, and there is often a lack of dissemination of replicable models in less digitally advanced contexts. This is particularly relevant in Italy, where the healthcare system is highly regionalized and heterogeneous, with 20 autonomous regional systems, fragmented EHR infrastructures, and significant disparities in digital adoption. According to the 7th GIMBE report (https://portale.fnomceo.it/presentato-l8-rapporto-gimbe-sul-servizio-sanitario-nazionale), regional differences exist in both health outcomes and ICT implementation, with only 42% of citizens consenting to access their electronic health records and large variations in EHR utilization across regions, highlighting the challenges of achieving real-time interoperability and data integration.

EmergInsight contributes to the literature in three main ways, and does so in order to address these specific gaps and challenges:

1. Complete functional integration: combines real-time monitoring, predictive analytics, and geospatial visualization in a modular, unified platform. This provides a simultaneous and consistent view of data, improving the ability to anticipate operational issues and support rapid decision-making.
2. Adaptation to fragmented ecosystems: it is designed to operate in contexts with heterogeneous data infrastructures, typical of the Italian healthcare system, demonstrating how interoperability can be achieved even in the absence of IT uniformity. This makes the model replicable in environments where other dashboards fail due to data fragmentation.
3. Dual operational and strategic purpose: it supports both daily emergency room decisions and regional planning and governance, providing an integrated approach between operational management and strategic decision-making processes. This allows data to be transformed from a mere description of the past into a proactive management and planning tool.

In this way, EmergInsight positions itself in the debate on dashboards for ED not only as a technical contribution, but as a concrete operational and strategic model, capable of combining immediate monitoring, predictive decision support, and territorial scalability. In other words, it shows how it is possible to overcome the limitations of fragmented contexts and provides a replicable example of integrated and proactive dashboards, useful for both daily activities and regional governance.

## Materials and methods

To test the platform and evaluate its effectiveness, data on visits to the emergency department of the San Giovanni Di Dio e Ruggi D’Aragona University Hospital for the years 2022-2023 were used. This choice was motivated by the completeness and quality of the available data, which allow for an accurate assessment of the platform’s performance in a real-world context characterized by complex variables and constantly evolving access flows.

The design of EmergInsight followed a clinician-led, iterative co-design process conducted jointly by emergency physicians, nurses, and operational managers from the San Giovanni di Dio e Ruggi d’Aragona University Hospital, together with data scientists and software engineers. A dedicated working group met on a biweekly basis over a six-month period to define clinical priorities, identify key performance indicators (KPIs), and review preliminary dashboard prototypes. During each iteration, mock-ups and live previews were tested directly in the ED environment using real anonymized data. Clinicians provided structured feedback on usability, visual clarity, and the relevance of displayed metrics—particularly those related to patient flow, waiting times, triage distribution, and Intensive Short Observation (OBI) (https://www.salute.gov.it/imgs/C_17_pubblicazioni_3142_allegato.pdf) utilization. Based on this feedback, the development team refined visualization layouts, color coding, and alert thresholds to ensure immediate interpretability in high-pressure contexts. Although the system builds on well-established ED metrics from the literature, these were customized to local workflows, data structures, and regional reporting needs, accounting for the fragmented data infrastructure typical of the Italian healthcare system. This iterative, practice-driven process ensured clinical relevance, operational feasibility, and interoperability, positioning EmergInsight as a replicable model for real-world emergency settings.

The data was extracted using custom SQL queries, run weekly and supported by automated daily update routines. This approach ensured both periodic data completeness and near real-time updates. The data was organized by year, quarter, and month in order to identify temporal patterns, including seasonal and periodic fluctuations in access. A multi-step data cleansing process was applied. Deduplication was performed using specific patient identifiers (eg, hash ID, date of birth, admission timestamp), consolidating records corresponding to the same clinical episode within a 24-hour interval. In the case of duplicate entries, only the first valid record was retained, based on triage consistency and hospital site. To handle missing data, imputation strategies were adopted: for categorical variables (eg, triage code, age groups), mode imputation was used, while for continuous variables (eg, length of stay in the OBI), median imputation was used, stratified by age and triage category. Variables with more than 10% missing data were excluded from inferential analyses, in line with methodological recommendations indicating that higher rates of missingness may introduce bias and reduce reliability

In relation to the geospatial analysis, anonymized information on patient residence (municipality/postcode) was linked to official administrative boundaries provided by ISTAT. Population data were used as denominators to calculate standardized visit rates per 100 000 residents and age-adjusted incidence by triage category. Aggregated indicators were generated daily and weekly to identify territorial disparities in demand, seasonal variations, and high-density clusters of ED access. Spatial computations were performed in R (with *sf* and *leaflet* packages) and cross-validated within the Qlik visualization layer to ensure consistency. The resulting maps were integrated into the dashboard, allowing users to filter data by time window, age group, and triage severity, and to visualize regional demand trends and catchment heterogeneity in real time.

To address the modular and interoperable architecture, the system was designed around three functional layers: (1) a data ingestion layer (SQL extraction, staging, and validation routines), (2) an analytics layer (statistical routines, KPI calculation, forecasting models, and spatial aggregation), and (3) a visualization layer (interactive dashboards and maps). Each module operates as an independent component connected through standardized data exchange formats (CSV/JSON) and an internal API structure, allowing individual modules to be updated or replaced without affecting the rest of the system. Interoperability across heterogeneous hospital data sources was achieved by implementing a standardized data dictionary mapping local variables to canonical ED indicators. Automated ETL processes ensured near real-time updates while preserving version control and data traceability. This modular structure not only supports scalability to multiple hospitals but also facilitates adaptation to different regional data environments with varying IT maturity levels. The AutoRegressive Integrated Moving Average (ARIMA) model^14^ was implemented to generate short-term forecasts of ED visits, supporting the platform’s objectives of optimizing operational efficiency and enabling proactive, data-driven resource allocation and patient flow management.

The resulting dataset was structured to support various analytical dimensions, including hospital site identification, distribution of visits by triage severity (white, green, yellow, and red), temporal distribution (daily, monthly, and quarterly), stratification by age group, weekend traffic, and OBI admissions. Several KPIs were calculated, such as the annual rate of emergency department visits per 100 000 residents, incidence by triage category, daily and monthly visit averages, and the specific weekend visit rate (Friday 6:00 pm–Monday 8:00 am). To facilitate modular analysis and visualization, the dataset was divided into thematic files, such as total annual visits, triage distribution, quarterly trends, and OBI admissions. Each file underwent a global consistency check to ensure structural integrity between variables and time periods, prioritizing critical fields such as access date, triage code, and patient age group. A standardized data dictionary (see Table 1) was also created to ensure clarity and interoperability, specifying variable names, data types, and transformation details, which facilitate the integration of the dataset into external statistical tools and business intelligence systems.

**Table 1.** Examples of variables included in the data dictionary.

Variable	Description	Type	Example
Access date	Date of access to the emergency room	Date (DD-MM-YYYY)	15-06-2023
Triage code	Triage codes classify the severity of patients’ conditions at ED arrival: Red**:** life-threatening emergencies, requiring immediate attention Yellow**:** urgent conditions, requiring prompt assessment Green**:** less urgent cases, manageable within standard waiting times White**:** non-urgent cases, minimal clinical risk	String	Green
Age group	Age range of the patient	String	65-79 years
Gender	Gender of the patient	String	Male
Weekend access	Access between Friday 18:00 and Monday 08:00	Boolean	1 (yes)
OBI admissions	Hospitalization in OBI	Boolean	1 (ie, yes)
Average OBI stay	Average duration in OBI (in days)	Numeric	2.5
Total visits	Total number of accesses in the period	Numeric	1500
Incidence rate	Rate per 100,000 population	Numeric	350

Further details on the infrastructure, including visual representations of the system architecture, the geospatial modules, and the interactive capabilities of the platform, can be found in the “Appendix A” (EmergInsight infrastructure details).

## Results

During the study period, the ED at “San Giovanni di Dio e Ruggi d’Aragona” University Hospital recorded a total of 114 050 visits, with 54 224 in 2022 and 59 826 in 2023. Seasonal variability was evident, with peaks in summer months. Most accesses were green codes (76.5% in 2022, 75.8% in 2023), followed by yellow (19.1% and 20.0%), white (2.3% and 1.8%), and red codes (2.0% and 2.2%). Time to first evaluation for red codes increased slightly from 26 min 25 s in 2022 to 32 min 44 s in 2023. Hospitalizations totaled 8236 and 8780, with an average admission rate of 15.1%, while abandonment rates decreased from 11.8% to 10.7%. Older adults (≥65 years) represented nearly 70% of OBI admissions, with an average stay of 2.4 days. Average waiting times at triage varied by code and year, and weekend accesses accounted for 32.6% of visits. Key descriptive statistics are summarized in Table 2.

**Table 2.** Summary of ED visits and operational metrics at “San Giovanni di Dio e Ruggi d’Aragona” University Hospital.

Metric	2022	2023	Notes/Units
Total ED visits	54 224	59 826	Number of visits
Seasonal peaks	July-August	July-August	Summer months
Triage distribution			Percentage of total visits
Green	76.5%	75.8%	–
Yellow	19.1%	20.0%	–
White	2.3%	1.8%	–
Red	2.0% (1111)	2.2% (1332)	Critical emergencies
Time to first evaluation (red codes)	26 min 25 s	32 min 44 s	–
Hospitalizations	8236	8780	–
Admission rate	15.1%	15.1%	Average across two years
Abandonment rate	11.8%	10.7%	Patients leaving before medical evaluation
Age distribution			Percentage of total visits
<65 years	31.2%		–
65-79 years	41.2%		–
≥80 years	27.6%		–
OBI admissions (≥65 years)	70%		Average OBI stay 2.4 days
Average waiting time at triage			hh: mm
Green	3:50	2:40	–
Yellow	1:40	1:42	–
White	1:29	5:32	–
Red	0:26	0:32	–
Weekend accesses	32.6%	32.6%	Approximately one-third of visits

The interactive dashboard, developed to optimize emergency care through real-time data visualization and analytics, is structured around several core modules that aim to improve operational efficiency and decision-making within EDs. Each module offers a unique set of visualizations and actionable insights to address different aspects of ED management.

### Operational metrics & regional trends

This module provides a high-level overview of KPIs, including total number of visits, average daily visits, average time spent in the emergency room, total number of hospital admissions, and overall incidence rate. These metrics provide immediate insight into patient volume and operational efficiency. In addition, the geographic distribution of emergency room visits is displayed via an interactive map, allowing administrators to identify regions with high patient demand or seasonal fluctuations. This visualization is critical for monitoring regional trends, ensuring adequate resource allocation, and predicting possible outbreaks or spikes in visits. By correlating access patterns with available resources, this module helps you make proactive, data-driven decisions about staffing and resource utilization.

**Figure 1.**
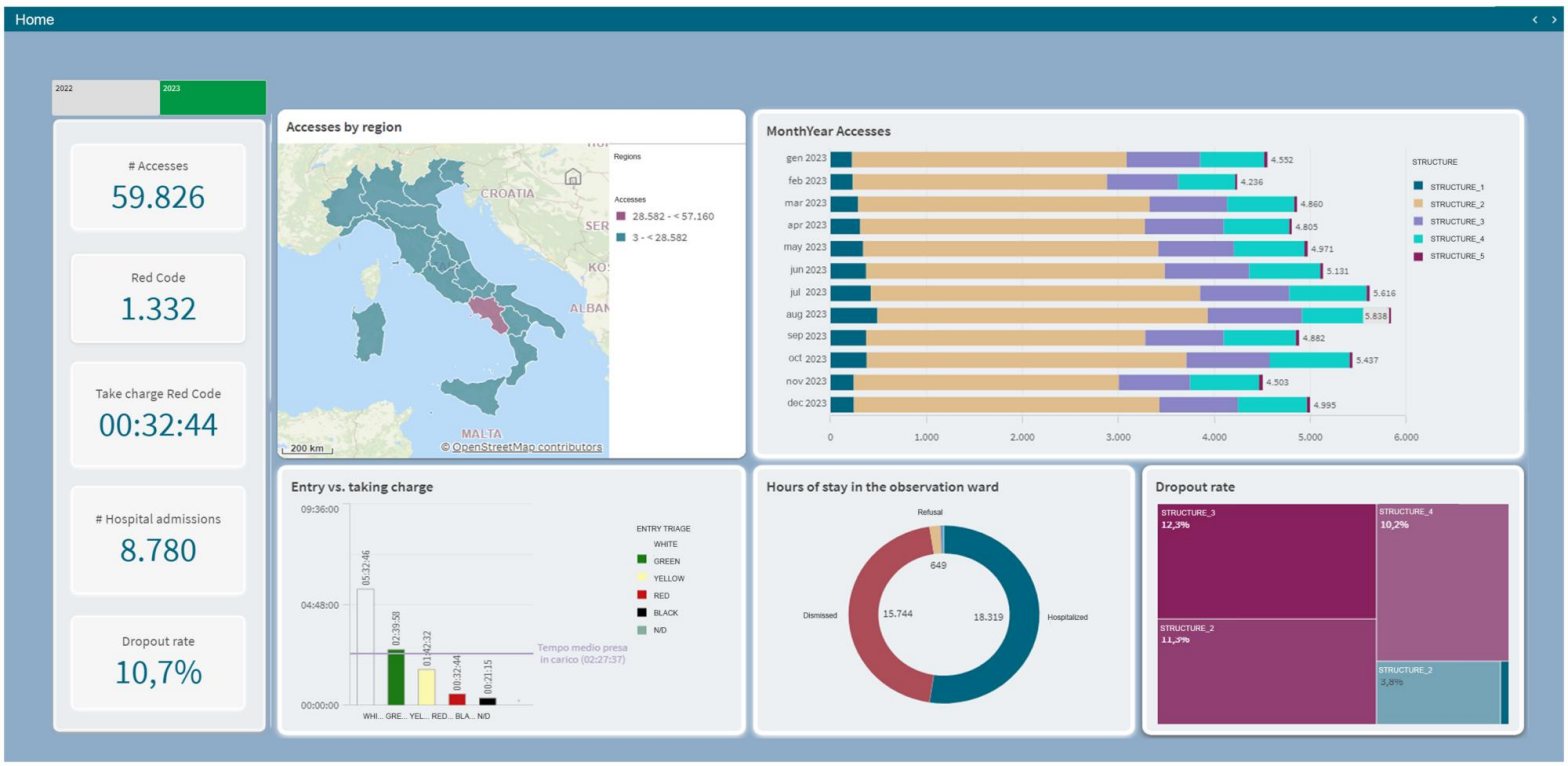
Overview of key operational metrics and geographic distribution of emergency department accesses.

Figure 1 provides a high-level overview of key operational metrics and the geographical distribution of emergency department accesses, offering administrators and clinicians a snapshot of the current situation and regional trends. The left-hand panel prominently displays essential KPIs such as total accesses, average daily accesses, average time spent in the ED, total hospital admissions, and the overall incidence rate. These metrics provide immediate quantitative context regarding the volume and flow of patients. The central top panel, a geographical map of Italy, visually represents the distribution of accesses across different regions. This type of visualization is crucial for identifying areas with high demand or potential outbreaks, supporting regional resource allocation and preparedness. The top right stacked bar chart details monthyear accesses, likely segmented by categories such as triage code or patient outcome, allowing for the identification of seasonal patterns, trends over time, and shifts in patient acuity or disposition. The bottom left bar chart, related to “Exit vs taking charge,” could illustrate the proportion of patients who leave before being seen versus those who complete their visit, a key indicator of patient satisfaction and potential bottlenecks. The bottom middle donut chart, showing the “Average stay in the observation unit,” provides insight into the utilization and efficiency of the OBI, a critical area for managing patients requiring extended monitoring. Finally, the bottom right treemap visualizes “Drop-out rates,” potentially broken down by factors like time of day, day of the week, or triage category, helping to pinpoint specific areas contributing to patients leaving prematurely.

### Patient acuity & temporal access patterns

This module provides insight into the severity of patients’ conditions and the timing of their visits. Using triage codes (white, green, yellow, and red), the module displays the distribution of patient severity, which is essential for ensuring that adequate staff and resources are allocated based on the severity of incoming patients. A trend graph tracks total monthly visits, helping to identify seasonal and hourly variations in demand. Understanding these trends allows for better forecasting of patient influx and helps administrators optimize staff schedules. Another key feature of this module is the breakdown of access data by age group, which provides detailed information on patient demographics and can help prioritize care for vulnerable populations, such as the elderly or people with chronic diseases.

**Figure 2.**
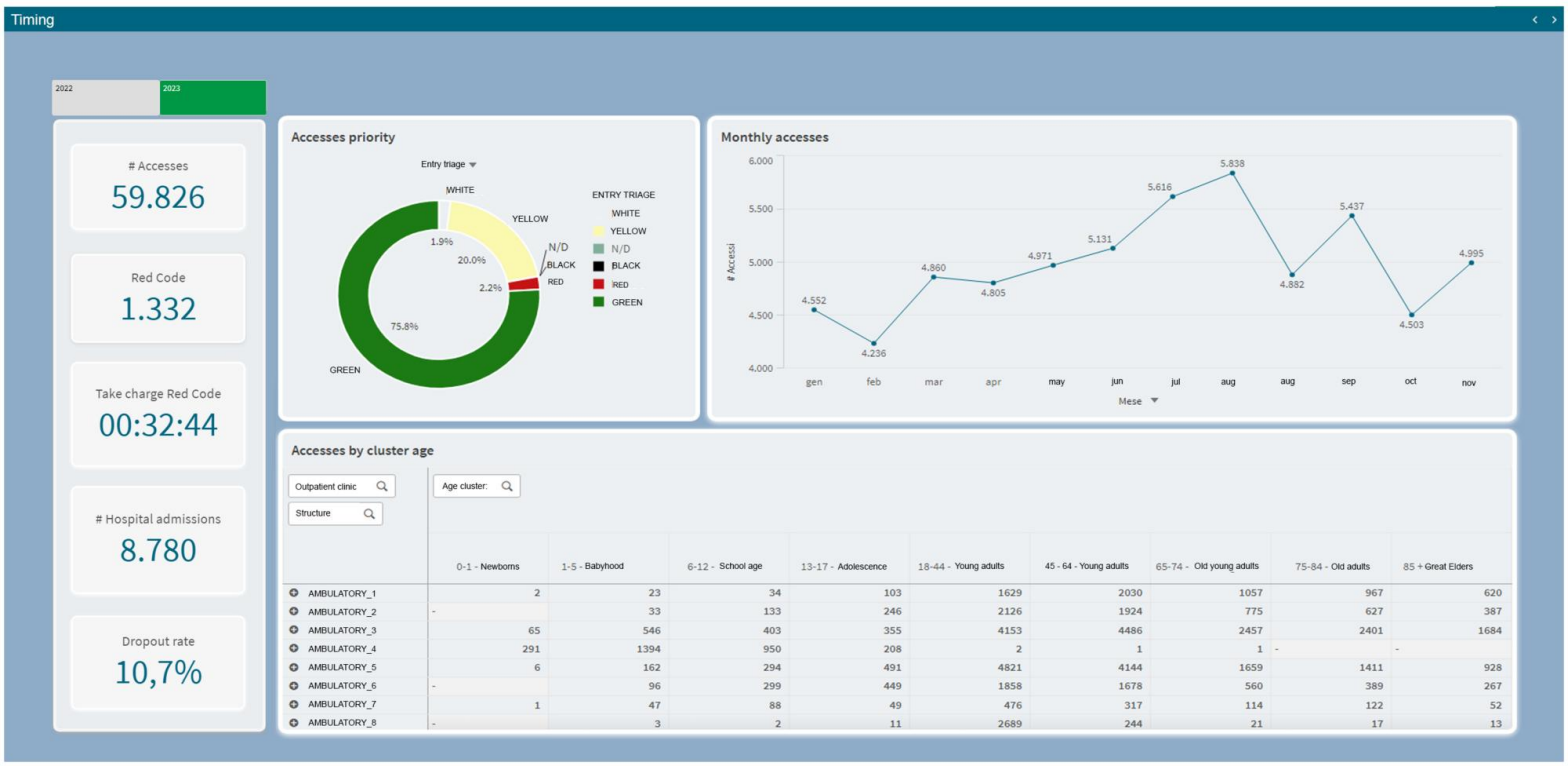
Detail of triage priorities and time trends of accesses. The figure includes key KPIs, a doughnut chart on the distribution of accesses by priority (color codes), a line graph on total monthly accesses, and a table or matrix on accesses stratified by age group.

Figure 2 delves deeper into patient acuity and temporal access patterns, providing crucial information for staffing and resource management. The consistent display of key performance indicators on the left ensures that core metrics remain visible regardless of the specific data view. The central top donut chart, “Accesses by priority,” offers a clear visual breakdown of patient volume by triage code (white, green, yellow, and red). This is fundamental for understanding the severity mix of patients presenting to the ED and for allocating clinical staff appropriately. The top right line chart, “Monthly accesses,” tracks the trend of total ED visits over a monthly period, enabling the identification of fluctuations and forecasting future demand. This temporal view is vital for strategic planning and resource allocation. The bottom panel, “Accesses by cluster age,” provides a detailed tabular or matrix view of patient accesses stratified by age groups. This is particularly useful for identifying vulnerable populations, understanding age-specific healthcare needs, and tailoring services accordingly. For example, a high volume of accesses in older age groups might indicate a need for specific geriatric care pathways within the ED.

### Patient outcomes & resource utilization

The patient outcomes module provides a detailed view of what happens to patients once they enter the emergency department. Key metrics include the percentage of patients admitted to the hospital, discharged, or transferred, helping administrators understand the downstream impact on hospital bed occupancy and overall patient flow through the system.

In addition, this module tracks the utilization of the OBI, a critical area for patients requiring extended monitoring. By displaying the frequency and duration of stays in the OBI, the system provides valuable insight into its capacity and efficiency, helping to prevent bottlenecks in patient care. The module also includes data on common medical conditions or major disorders, allowing healthcare professionals to identify recurring trends or issues that may require targeted intervention. In particular, by combining data on patient outcomes and resource utilization, the dashboard allows you to identify which departments or areas of care are under the most stress, where performance is declining, or where improvements are being made. This information enables timely operational interventions, such as reassigning staff, optimizing patient flow, or prioritizing interventions in specific departments, thereby promoting both efficiency and quality of care.

**Figure 3.**
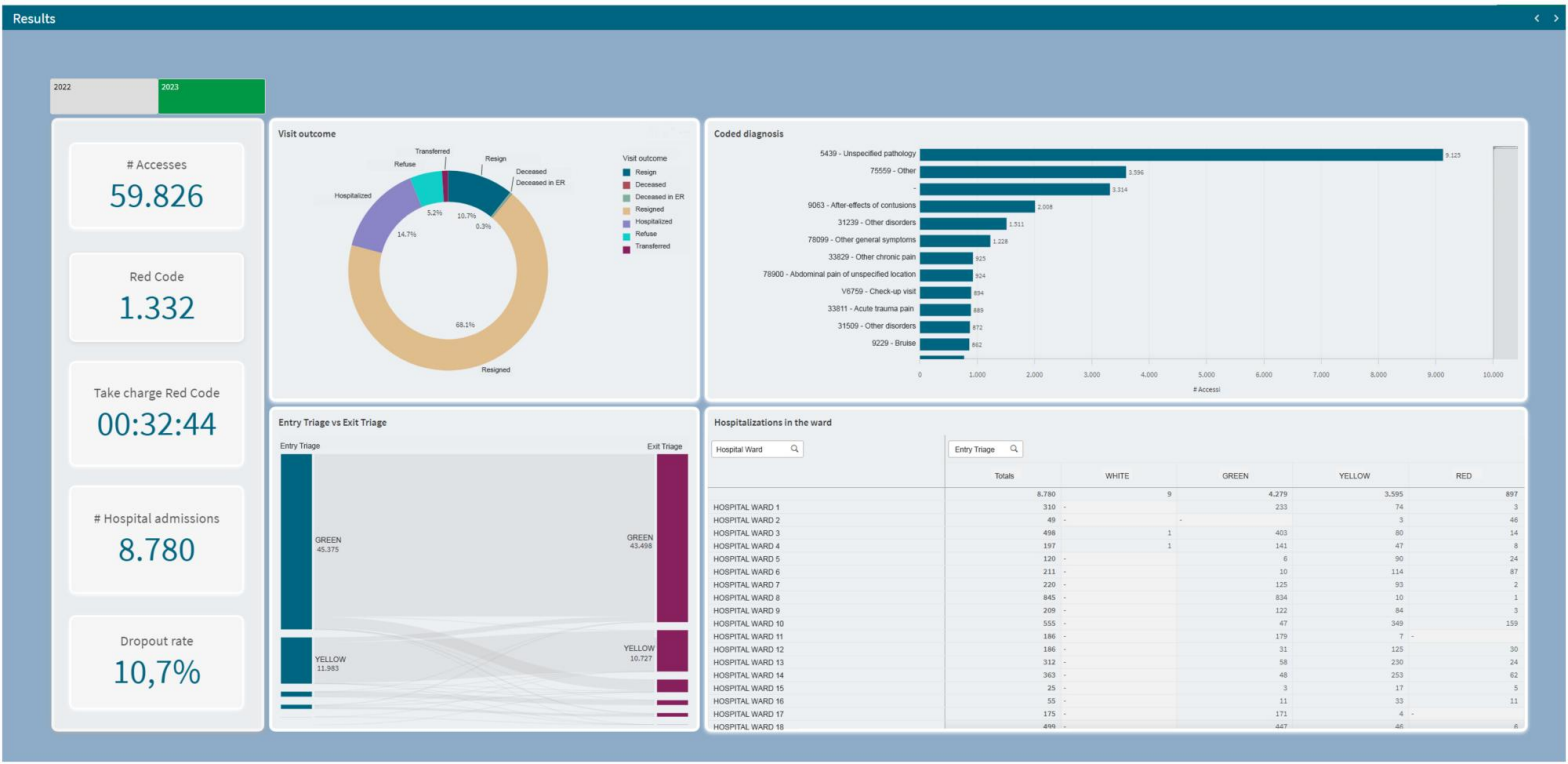
More details on patient outcomes and OBI utilization. The figure presents key KPIs, a doughnut chart on admissions (post-ED outcomes), a horizontal bar graph on “Solid Impact” (possible medical conditions or reasons for access), a bar graph on “OBI hospitalization,” and a “Performance & Trend” panel with detailed metrics and time trends.

Figure 3 offers further granularity on patient outcomes and the utilization of specific resources like the OBI, supporting clinical and operational decision-making. The standard display of KPIs on the left provides ongoing context. The central top donut chart, “Admissions,” visualizes the disposition of patients after their ED visit, showing the proportion admitted to the hospital versus discharged or transferred. This metric is a key indicator of the severity of presenting conditions and the downstream impact on hospital bed availability. The top right horizontal bar chart, “Solid Impact,” likely represents the frequency or impact of different medical conditions or chief complaints, offering insights into the most common reasons for ED visits and potential areas for targeted interventions or public health initiatives. The bottom left bar chart, “OBI Hospitalization,” provides specific data on admissions to and potentially the duration of stay within the OBI, allowing for focused analysis of this resource-intensive area. The bottom right panel, “Performance & Trend,” is likely a table or chart presenting detailed performance metrics and their trends over time. This could include metrics such as waiting times per triage category, length of stay, or throughput times, providing the data necessary to identify areas for process improvement and track the effectiveness of implemented changes.

### Forecasting & predictive analytics

One of the most powerful features of the dashboard is its predictive capability, made possible by the ARIMA model. By analyzing historical patterns of emergency room visits, including daily, weekly, and seasonal trends, as well as the effects of holidays and weekends, the model generates short-term forecasts. These forecasts provide hospital administrators with useful information that goes beyond simple reporting: they enable the emergency department to anticipate periods of high patient influx, plan staff shifts accordingly, and ensure that critical resources, such as beds, monitoring equipment, and specialized personnel, are available when and where they are most needed. For example, the model can predict potential spikes in severe cases, such as patients with yellow or red triage, enabling proactive adjustments in staffing and preparation of care spaces. Similarly, it can anticipate expected increases in OBI admissions, helping to prevent bottlenecks and ensuring continuous patient monitoring without compromising the quality of care. By integrating these forecasts with real-time metrics on patient severity, demographics, and regional demand patterns, the ARIMA module becomes a practical decision support tool that directly contributes to operational efficiency and improved patient flow management. In addition to immediate operational benefits, predictive insights also support strategic planning. Trends identified by the ARIMA model can guide decisions related to resource allocation across hospital locations, temporary staff reassignments during seasonal peaks, and long-term capacity planning. The integration of predictive analytics within the interactive dashboard ensures that the model’s results are not static: they are continuously updated as new data arrives, allowing administrators to respond dynamically to changing conditions in the emergency room. For a concrete example of how the ARIMA model is applied to short-term forecasting of daily emergency department visits, see “Appendix B” Performance and diagnostic evaluation of the ARIMA model for short-term forecasting of daily emergency department visits.

## Discussion

One of the most pressing issues in Italy is the fragmentation of healthcare data, both between regions and within institutions. The lack of standardized exchange protocols and the coexistence of multiple separate EHR systems limit the possibility of building a unified view of the patient, undermining the potential of real-time analysis. Healthcare professionals, such as emergency physicians and nurses, often work in high-pressure environments with heavy workloads and limited time for training, which can cast doubt on the effectiveness of new technologies. Resistance to change is further exacerbated by the perception that learning new tools is a distraction from established practices, despite the potential benefits in terms of efficiency and quality of care.^15^^,^^16^

The implications of this work extend beyond the pilot site, addressing broader systemic needs. At the regional level, dashboards can support peak capacity planning, epidemic response, and equitable resource distribution. At the hospital level, they offer tools to identify bottlenecks in processes and optimize staff distribution. At the national level, platforms such as ours could improve preparedness and resilience, especially in the management of mass or seasonal events. In addition, the system’s ability to generate aggregated and anonymized data streams facilitates public health research, policy evaluation, and performance benchmarking, key pillars of a learning-based healthcare system. In addition to real-time operational capabilities, the platform includes advanced tools for retrospective analysis of historical data, enabling the identification of long-term trends and the evaluation of the effectiveness of targeted organizational interventions. The ability to compare performance indicators across institutions or time periods provides a strategic advantage for evidence-based decision-making at both the micro-and macro levels of emergency management.

It is important to emphasize that the proposed prototype offers a concrete vision of rapid emergency preparedness and response, positioning dashboards as real-time decision support systems to improve situational awareness and coordinate the system during critical events. By providing immediate access to crucial data and analysis, these dashboards can significantly improve the speed and accuracy of decision-making, especially in situations that require a timely response, such as natural disasters, pandemics, or large-scale accidents. Future developments will focus on expanding these capabilities by incorporating advanced predictive models, real-time data from wearable devices, and integration with the Internet of Medical Things (IoMT). Specifically, the use of advanced models such as Long Short-Term Memory (LSTM) and Gated Recurrent Units (GRU) will allow the system to learn complex temporal dependencies and nonlinear relationships within the emergency flow data, improving the accuracy of short- and medium-term forecasts compared to the current ARIMA approach. In parallel, ensemble learning techniques such as XGBoost and LightGBM will be explored to integrate multiple sources of information—including clinical parameters, demographic variables, and historical access patterns—enabling more precise predictions of overcrowding risks and hospital admissions.

Real-time data from wearable devices, such as continuous monitoring of heart rate, oxygen saturation, and activity levels, will feed into the dashboard through IoMT integration, allowing early detection of clinical deterioration before the patient arrives at the emergency department. This capability will transform the system from a reactive tool into a proactive monitoring environment that supports early intervention and optimizes resource allocation.

Finally, the integration of generative artificial intelligence tools—including large language models capable of processing unstructured clinical data—will make it possible to extract and synthesize meaningful information from triage notes, medical records, and free-text observations. This will enrich the analytical depth of the platform, enabling it to provide context-aware insights and to support clinical and organizational decisions based on both structured and narrative data.

## Supplementary Material

ooag008_Supplementary_Data

## Data Availability

The datasets used and analyzed during the current study are not publicly available due to data protection and institutional policies. However, anonymized summary data and aggregated metrics are available from the corresponding author upon reasonable request and subject to data sharing agreements with the Azienda Ospedaliera Universitaria ‘San Giovanni Di Dio e Ruggi D’Aragona’.
