# Supplementary material for: EmergInsight: a real-time dashboard for optimizing emergency care through data visualization and analytics
Source: JAMIA Open. 2026 Jan 20;9(1):ooag008. doi: 10.1093/jamiaopen/ooag008 (PMC12824725; doi:10.1093/jamiaopen/ooag008)
Supplement: ooag008_Supplementary_Data [file ooag008_supplementary_data.docx]

**EmergInsight: A Real-Time Dashboard for Optimizing Emergency Care through Data Visualization and Analytics**

Authors: **Francesco Branda^1,^ PhD, Vincenzo Andretta^2^ PhD, Mohamed Mustaf Ahmed^3,*^ PhD, Antonio Rizzelli^4^ Mr, Valentina Cerrone^5^ MSN, Giovanni Boccia^2^ PhD, Giancarlo Ceccarelli^6^ PhD, Fabio Scarpa^7^ PhD, Massimo Ciccozzi^1^ PhD**

Affiliations

1. Unit of Medical Statistics and Molecular Epidemiology, University Campus Bio-Medico of Rome, Rome, Italy
2. Department of Medicine, Surgery and Dentistry - Salerno Medical School, University of Salerno, Fisciano, Italy
3. Faculty of Medicine and Health Sciences, SIMAD University, Mogadishu, Somalia
4. Research and Development Manager and Qlik Specialist, Ethica System Srl, Capurso, Italy
5. University Clinical Oncology Unit, University Hospital ‘San Giovanni Di Dio e Ruggi D’Aragona’, Salerno, Italy
6. Department of Public Health and Infectious Diseases, University of Rome Sapienza, Rome, Italy
7. Department of Biomedical Sciences, University of Sassari, Sassari, Italy

*Corresponding author: Mohamed Mustaf Ahmed, Faculty of Medicine and Health Sciences, SIMAD University, Mogadishu, Somalia (momustafahmed@simad.edu.so). ORCID: <https://orcid.org/0009-0006-5991-4052>

## **Appendix A**

## **EmergInsight infrastructure details**

The EmergInsight platform is built on the Qlik Sense Cloud infrastructure, combining advanced computational capabilities with a fully scalable microservices architecture orchestrated via Kubernetes, as shown in Figure S1. This design enables real-time, interactive exploration of heterogeneous healthcare data while maintaining high fault tolerance, minimal latency (<2 sec), and near-continuous availability (99.995% uptime). At the core, the Qlik Associative Engine leverages in-memory processing to dynamically link data across multiple sources, eliminating the need for predefined queries. This allows clinicians and administrators to navigate seamlessly between clinical, operational, and logistical data, identify correlations between triage codes, patient flow, resource utilization, and detect abnormal trends in real time.

Data integration is achieved through specialized connectors and a Python- and Kafka-based pipeline, capable of processing up to 10,000 events per second. Sources include hospital EHRs via HL7/FHIR adapters, IoT devices for real-time vital signs, and legacy operational systems via REST APIs, all harmonized into a flexible MongoDB schema capturing pseudonymized patient identifiers, timestamps, triage codes, structured clinical data (vital signs, ICD-11 diagnoses), and operational metadata (resources used, care pathways). Columnar compression reduces the memory footprint by approximately 70% compared to traditional databases, while dynamic associations between clinical and logistical data enable flexible and real-time calculation of KPIs. Among these, the *LWBS rate* measures the percentage of patients who leave without being seen and is defined as:

$$LWBS rate=\frac{number of patients who left without being seen}{total number of patients who arrived}\times100$$

Similarly, the *Door-to-Doctor time* quantifies the median time elapsed between a patient's arrival and the moment they are first seen by a doctor:

$$Door-to-Doctor time: Median (Time doctor sees patient) - (Patient arrival time)$$

Predictive analytics are implemented using AutoRegressive Integrated Moving Average (ARIMA) models, a class of statistical time series models that combine three components: autoregression (AR), which models the dependency of the current value on its past values; integration (I), which accounts for non-stationarity by differencing the series; and moving average (MA), which models the dependency on past forecast errors. These models are well-suited for capturing trends, short-term fluctuations, and seasonality in daily ED visit counts from January 2022 to December 2023. The optimal ARIMA(1,1,2)(2,0,0)configuration was selected using R’s auto.arima() function, which searches over possible ARIMA orders and seasonal components based on information criteria such as the Akaike Information Criterion (AIC). Model validation included inspection of residual autocorrelations via the autocorrelation function (ACF), assessment of residual normality using Q-Q plots, and evaluation of residual variance with summary statistics. Weekly seasonality was included by specifying a frequency of seven in the time series, allowing the model to capture recurring weekly patterns and short-term fluctuations in ED visits. Forecast accuracy was quantified on a 30-day out-of-sample test set using standard metrics:

- **Mean Absolute Percentage Error (MAPE):** $\text{MAPE}=\frac{1}{n}\sum_{t=1}^{n} \mid\frac{y_{t}-\hat{y}_{t}}{y_{t}}\mid\times100$ , where $y_{t}$are observed values and $\hat{y}_{t}$are predictions.
- **Mean Absolute Error (MAE)**: $\text{MAE}=\frac{1}{n}\sum_{t=1}^{n} \mid y_{t}-\hat{y}_{t}\mid$, giving the average deviation in absolute counts.
- **Root Mean Square Error (RMSE)**: $\text{RMSE}=\sqrt{\frac{1}{n}\sum_{t=1}^{n} (y_{t}-\hat{y}_{t})^{2}}$, penalizing larger errors more heavily.
- **Coefficient of determination (R²)**: $R^{2}=1-\frac{\sum(y_{t}-\hat{y}_{t})^{2}}{\sum(y_{t}-y)^{2}}$, indicating the proportion of variance explained by the model.

Forecast accuracy is often expressed as $100-\text{MAPE}$, giving the percentage of correctly predicted values relative to observed counts, and forecasts are produced for 30-day horizons and updated daily within the dashboard, providing real-time operational insights that enable ED staff to anticipate patient surges, optimize staffing, and implement contingency protocols proactively.

The dashboards support self-service analytics, interactive drill-downs from aggregate KPIs to individual patient cases, geospatial mapping to identify high-demand areas, and alert systems for early warnings. Advanced features include cognitive search over unstructured clinical notes, AI-based insight recommendations, and mobile access for offline analytics. Security and governance are enforced through role-based access control (RBAC), dynamic data masking, AES-256 encryption with AWS KMS key management, pseudonymization following FPE standards, and blockchain-based immutable audit logs, ensuring compliance with GDPR and certifications including ISO 27001/27017/27018 and HITRUST CSF.

Finally, the elastic cloud design supports auto-scaling up to 1,000 concurrent users, intelligent load balancing, failover with downtime under 15 seconds, continuous deployment without service interruption, and rollback/A-B testing for new features. This architecture enables proactive operational management, flexible integration of new data parameters without downtime, and supports future expansions such as AI-driven semantic analysis of clinical notes, augmented reality visualizations, and blockchain-based data federation across hospitals.


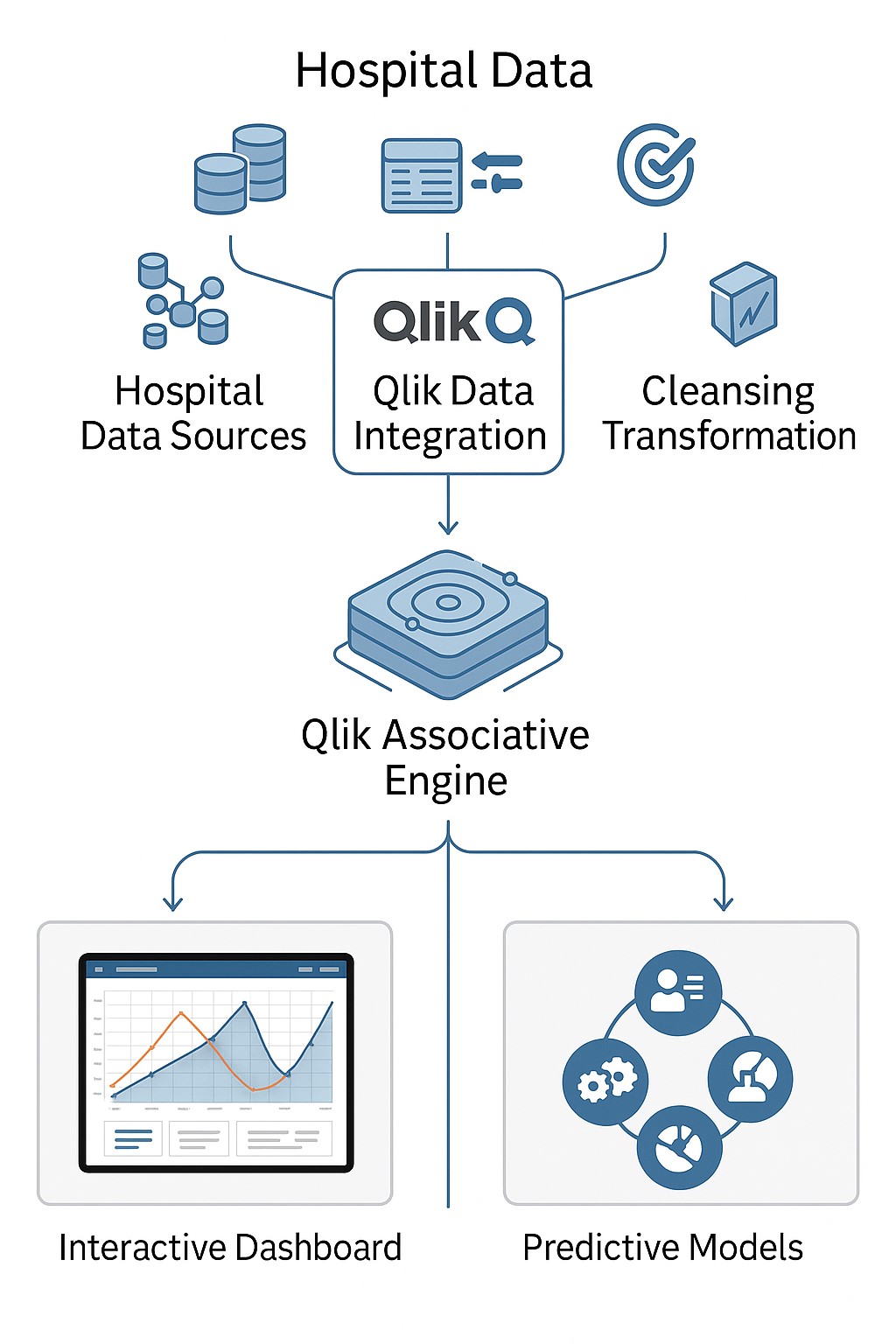


**Figure S1.** Architecture of the EmergInsight platform.

## **Appendix B**

**Performance and diagnostic evaluation of the ARIMA model for short-term forecasting of daily emergency department visits**

Figure S2 illustrates the performance and diagnostic assessment of the ARIMA model for forecasting daily ED visits. Over a 30-day out-of-sample period, the model achieved an R² of 0.61, indicating that 61% of the variance in daily ED visits is explained. The MAE was 22.77 visits per day, the RMSE was 29.49 visits per day, and the MAPE was 7.37%, highlighting that the model delivers reliable short-term predictions with average deviations well below 8% of observed daily counts. Figure S2A illustrates the observed and predicted daily ED visits over the 30-day forecast horizon. Observed values are shown in blue, ARIMA predictions in red, and the 95% confidence interval is represented by a shaded area, reflecting the uncertainty bounds of the forecasts. The comparison shows that the model effectively captures both baseline trends and short-term fluctuations in patient arrivals, with the predicted values closely following the observed patterns [1,2].

Figure S2B presents diagnostic plots for the residuals of the ARIMA model. The top left panel shows residuals over time, which are scattered randomly around zero with a mean of 1.16 and a standard deviation of 29.49, ranging from -88.99 to 126.85, indicating no systematic bias. The top right panel displays a histogram of residuals, showing an approximately normal distribution. The bottom left panel shows a Q-Q plot, confirming that residual quantiles align well with a theoretical normal distribution. The bottom right panel shows the autocorrelation function (ACF) of the residuals; most autocorrelation values are below the 95% significance threshold, with only lag 2 exceeding it, indicating minimal remaining autocorrelation [1,3].

Additional statistical analyses were performed to further assess residual properties. A Shapiro–Wilk test indicated slight deviation from normality (W = 0.990, p < 0.001), which is expected for count-based data but does not compromise predictive reliability. The Ljung–Box test confirmed the absence of significant autocorrelation in the residuals (X² = 27.38, df = 20, p = 0.125), and the Augmented Dickey–Fuller test demonstrated stationarity (Dickey–Fuller = -8.5445, p ≈ 0.01), indicating that the model adequately removed trends and seasonal patterns. Together, these diagnostics support the robustness of the ARIMA model for short-term forecasting of ED visits, with residuals showing near-random behavior, minimal autocorrelation, and stationarity, reinforcing confidence in its operational use for resource planning and surge preparedness in the emergency department [2-4]. Together, these diagnostic results support the adequacy of the ARIMA model and its suitability for short-term forecasting of ED visits. The results demonstrate that the model provides accurate predictions with residuals exhibiting near-random behavior, normality, and minimal autocorrelation, reinforcing confidence in its operational use for resource planning and surge preparedness in the emergency department.

**Figure S2. A.** Forecasting daily ED visits using ARIMA. The blue line represents observed visits, the red line shows ARIMA predictions, and the shaded area indicates the 95% confidence interval. **B.** Diagnostic plots for ARIMA model residuals. The top left panel shows residuals over time with a random scatter around zero. The top right panel displays a histogram of residuals indicating an approximately normal distribution. The bottom left panel presents a Q-Q plot comparing residual quantiles to the theoretical normal distribution, and the bottom right panel shows the ACF plot of residuals with no significant autocorrelation beyond lag 0.

**References**

1. Hyndman RJ, Athanasopoulos G. Forecasting: principles and practice. OTexts; 2018 May 8.
2. Box GE, Jenkins GM, Reinsel GC, Ljung GM. Time series analysis: forecasting and control. John Wiley & Sons; 2015 May 29.
3. Shumway RH, Stoffer DS. Time series analysis and its applications: with R examples. New York, NY: Springer New York; 2006 May.
4. Hyndman R, Koehler A, Ord K, Snyder R. Forecasting with exponential smoothing: the state space approach. Berlin, Heidelberg: Springer Berlin Heidelberg; 2008 Jun 19.
